# Molecular dynamics simulations give insight into d-glucose dioxidation at C2 and C3 by *Agaricus meleagris* pyranose dehydrogenase

**DOI:** 10.1007/s10822-013-9645-7

**Published:** 2013-04-17

**Authors:** Michael M. H. Graf, Urban Bren, Dietmar Haltrich, Chris Oostenbrink

**Affiliations:** 1 Food Biotechnology Laboratory, Department of Food Science and Technology, University of Natural Resources and Life Sciences (BOKU), Muthgasse 18, 1190 Vienna, Austria; 2 Institute of Molecular Modeling and Simulation, University of Natural Resources and Life Sciences (BOKU), Muthgasse 18, 1190 Vienna, Austria; 3 Laboratory for Molecular Modeling, National Institute of Chemistry, Hajdrihova 19, 1001 Ljubljana, Slovenia

**Keywords:** Flavoproteins, Protein–ligand interactions, Reaction mechanism, Enzyme promiscuity, Bioelectrochemistry, GROMOS

## Abstract

The flavin-dependent sugar oxidoreductase pyranose dehydrogenase (PDH) from the plant litter-degrading fungus *Agaricus meleagris* oxidizes d-glucose (GLC) efficiently at positions C2 and C3. The closely related pyranose 2-oxidase (P2O) from *Trametes multicolor* oxidizes GLC only at position C2. Consequently, the electron output per molecule GLC is twofold for PDH compared to P2O making it a promising catalyst for bioelectrochemistry or for introducing novel carbonyl functionalities into sugars. The aim of this study was to rationalize the mechanism of GLC dioxidation employing molecular dynamics simulations of GLC–PDH interactions. Shape complementarity through nonpolar van der Waals interactions was identified as the main driving force for GLC binding. Together with a very diverse hydrogen-bonding pattern, this has the potential to explain the experimentally observed promiscuity of PDH towards different sugars. Based on geometrical analysis, we propose a similar reaction mechanism as in P2O involving a general base proton abstraction, stabilization of the transition state, an alkoxide intermediate, through interaction with a protonated catalytic histidine followed by a hydride transfer to the flavin N5 atom. Our data suggest that the presence of the two potential catalytic bases His-512 and His-556 increases the versatility of the enzyme, by employing the most suitably oriented base depending on the substrate and its orientation in the active site. Our findings corroborate and rationalize the experimentally observed dioxidation of GLC by PDH and its promiscuity towards different sugars.

**Electronic supplementary material:**

The online version of this article (doi:10.1007/s10822-013-9645-7) contains supplementary material, which is available to authorized users.

## Introduction

The sugar oxidoreductase pyranose dehydrogenase (PDH, EC 1.1.99.29) is a monomeric flavoprotein of approximately 65 kDa containing ~7 % carbohydrates. PDH is found in *Agaricaceae* and *Lycoperdaceae,* which represent a small group of fungi involved in lignocellulose breakdown from forest litter [1]. The enzyme was first isolated and characterized from *Agaricus bisporus* [2], and subsequently from *Macrolepiota rhacodes* [3], *Agaricus xanthoderma* [4] as well as *Agaricus meleagris* [1]. PDH from *A. meleagris* has been studied in most detail to date with respect to its biochemical properties and potential applications [5].

At commencement of this work, a preliminary version of the high resolution (1.6 Å) X-ray crystal structure of *A. meleagris* PDH with PDB code 4H7U was kindly made available to us [6]. Together with glucose oxidase (GOX, EC 1.1.3.4), the flavin domain of cellobiose dehydrogenase (CDH, EC 1.1.99.18), and pyranose 2-oxidase (P2O, EC 1.1.3.10), PDH belongs to the structural family of glucose–methanol–choline–oxidoreductases (GMC oxidoreductases) [5]. PDH exhibits broad carbohydrate substrate specificity compared to other GMC oxidoreductases. Depending on the sugar-substrate, it can perform (di)oxidations at C1, C2, C3, or C4 positions of the hexapyranose ring [5, 7]. This property, however, depends strongly on the source of the enzyme as well as on the sugar substrate. For example, d-glucose (GLC) is (di)oxidized at positions C2 and C3 by PDH from *Agaricus* spp. but only at position C3 by PDH from *M. rhacodes*, while P2O oxidizes this sugar almost exclusively at C2 [8]. Consequently, the electron output per molecule of GLC is twofold for *Agaricus* PDH compared to P2O. This makes PDH a promising catalyst for applications in bioelectrochemistry or for introducing novel carbonyl functionalities into sugars [5].

In order to investigate the PDH–GLC interactions, molecular dynamics (MD) simulations were performed. MD simulations are becoming an increasingly popular standard tool in biosciences to explore dynamic system properties or to investigate features not readily accessible by experimental means. Because of this, MD simulations and experiments represent complementing methods to study different aspects of nature [9, 10].

**Fig. 1:**
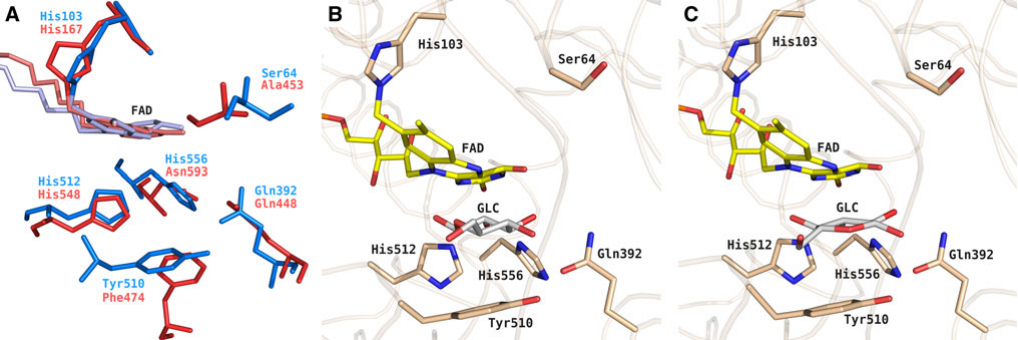
Active site structures of homotetrameric pyranose 2-oxidase (P2O) from *Trametes multicolor* and monomeric pyranose dehydrogenase (PDH) from *Agaricus meleagris*. **a** Superposition of active site residues from PDH, *blue* (PDB: 4H7U) and P2O, *red* (PDB: 3LSK). For clarity, the FAD moiety was colored *light blue* in PDH or *light red* in P2O and the atoms of the FAD ribityl side chains were omitted. **b**, **c** After superpositioning, d-glucose coordinates were grafted from the P2O structure with PDB accession codes (**b**) 3PL8 or (**c**) 2IGO into PDH. For clarity, P2O is not shown. Atom-coloring scheme: carbon (*beige*, protein; *yellow*, FAD; *white*, ligand), nitrogen (*blue*), oxygen (*red*), and phosphate (*orange*). Figures were generated using PyMOL (http://www.pymol.org/)

Unfortunately, no experimentally determined structure of any PDH-substrate complex is currently available. Hence the only possibility to study the detailed interactions of PDH with its major substrate GLC is by computational means. Although the GMC oxidoreductases PDH and P2O (PDB: 3LSK) [11] possess a relatively low overall sequence identity of 16 %, their sugar-binding sites are very similar as demonstrated by superposition in Fig. 1a with an atom-positional root-mean-square deviation (RMSD) of 0.13 nm for all heavy atoms in this figure. Moreover, experimentally determined P2O structures with GLC bound in two different orientations are available in the PDB: 3PL8 [12] and 2IGO [8]. Therefore, these two P2O–GLC structures were used to retrieve the coordinates of two PDH–GLC complexes for this study. After initial superposition, the GLC coordinates were manually grafted into the PDH structure in an orientation according to the P2O-PDB code 3PL8 (Fig. 1b) [12]—termed pose A—or according to the P2O-PDB code 2IGO (Fig. 1c) [8]—termed pose B.

In contrast to P2O, PDH contains two potentially catalytic residues, His-512 and His-556, in the active site (Fig. 1a). In the present study, extensive MD simulations were conducted of PDH in its apo form, of GLC (d-glucose), and of a variety of complexes between the two, differing in the poses of the substrates and in the protonation state of the active site histidines. The results were compared in terms of important interactions, conformational entropies and interaction energies between PDH and GLC, and the structural dynamics and stability was followed in detail. An atomistic understanding of these properties will provide detailed insights into PDH–GLC interactions, which immediately suggest future site-directed mutagenesis experiments and can ultimately pave the way towards the desired bioelectrochemical applications.

## Methods

### Preparation of initial structures

A preliminary version of the crystal structure of *A. meleagris* PDH at 1.6 Å resolution with PDB entry code 4H7U without bound GLC served as a starting point [6]. In this structure, the isoalloxazine ring of the flavin is modified by a covalent monoatomic oxygen species at position C(4a). Since PDH does not react with oxygen under standard reaction conditions, this monoatomic oxygen species is most likely a result of oxygen radicals present during X-ray data collection and was therefore not considered for subsequent MD simulations. This structure does not contain the 25 amino acid long signal sequence (MLPRVTKLNSRLLSLALLGIQIARG), which is cleaved off during secretion. Under physiological conditions, PDH is a glycosylated protein and sugar moieties are observed bound to Asn-75 and Asn-294, which were verified as potential glycosylation sites using the program NetNGlyc 1.0 [13]. In the current work, the sugar moieties NAG-901 (covalently attached to Asn-75), NAG-902 (covalently attached to Asn-294), and NAG-903 (covalently attached to NAG-902) were removed from the structure. Phosphate ion PO4-910, most likely a crystallization-buffer artifact surrounded by amino acid residues Arg-87 to Asp-90 and Pro-406 to Lys-407, was removed as well. The amino and carboxy termini were charged; all arginines, cysteines and lysines were protonated, and all aspartates and glutamates were deprotonated. Three different protonation states of His-512 and His-556 in the active site were considered: (1) His-512 being fully protonated and His-556 in its neutral state (proton at N_ε_), from now on labeled as PN; (2) His-512 in its neutral state (proton at N_δ_) and His-556 fully protonated, from now on labeled as NP; (3) both His-512 and His-556 fully protonated, from now on labeled as PP. The selection of the tautomeric states for neutral histidines was such that in the x-ray structure, the deprotonated nitrogen atoms pointed towards the active site. All remaining histidines were doubly protonated with exception of His-103, which is covalently bound to the FAD and was N_δ_-protonated. Two different substrate poses in the protein-substrate complex were generated: (1) Pose A: PDH alignment with P2O in complex with 3-fluoro-3-deoxy-d-glucose (PDB: 3PL8) [12]; (2) Pose B: PDH alignment with P2O in complex with 2-fluoro-2-deoxy-d-glucose (PDB: 2IGO) [8] (Fig. 1b, c). In all systems, the sugar coordinates were grafted into the active site of PDH after superposition and the fluorine of the sugar was replaced by a hydroxyl group. The combination of three protonation states and two substrate poses led to the definition of six protein-substrate complex systems. In addition, the apo protein was simulated (system PDH) using protonation state PP and system GLC was prepared consisting of β-d-glucose with coordinates taken from P2O-PDB 2IGO [8]. Table 1 gives an overview of all simulated systems.

**Table 1:** Overview of simulated systems

Code	Protonation (His-512/His-556)	Ligand	Water molecules	Sodium ions	Total number of atoms	Runs
GLC^a^		d-glucose^b^	1,162	–	3,503	1
PN_A	+/0	d-glucose^c^	13,202	5	45,289	2
PN_B	+/0	d-glucose^b^	13,212	5	45,319	2
NP_A	0/+	d-glucose^c^	13,198	5	45,277	2
NP_B	0/+	d-glucose^b^	13,203	5	45,292	2
PP_A	+/+	d-glucose^c^	13,187	4	45,244	2
PP_B	+/+	d-glucose^b^	13,210	4	45,313	4
PDH	+/+	–	13,434	4	45,968	1

^a^ d-glucose free in solution

^b^ d-glucose coordinates according to PDB code 2IGO

^c^ d-glucose coordinates according to PDB code 3PL8

### Simulation setup

All MD simulations were carried out employing the GROMOS11 software package [14] with the 53A6 force field [15]. His-103 and FAD were covalently bound to each other and their force field parameters and topologies were adapted accordingly. The four studied systems were energy-minimized in vacuo using the steepest-descent algorithm: first, the sugar atoms were energy minimized with constrained PDH coordinates, and second, both the sugar- and the PDH atoms were energy minimized. Each energy-minimized system was placed into a periodic, pre-equilibrated, and rectangular box of SPC water [16]. Minimum solute to box-wall and minimum solute to solvent distances were set to 0.8 and 0.23 nm, respectively. To relax unfavorable atom–atom contacts between the solute and the solvent, energy minimization of the solvent was performed while keeping the solute positionally restrained using the steepest-descent algorithm. Finally, four to five water molecules that had the most favorable electrostatic potential for replacement by a positive ion were replaced by sodium ions to achieve electroneutrality in the protein systems.

For the equilibration, the following protocol was used: initial velocities were randomly assigned according to a Maxwell–Boltzmann distribution at 50 K. All solute atoms were positionally restrained through a harmonic potential with a force constant of 2.5 × 10^4^ kJ mol^−1^ nm^−2^ not to disrupt the initial conformation, and the systems were propagated for 20 ps. In each of the five subsequent 20 ps MD simulations, the positional restraints were reduced by one order of magnitude and the temperature was increased by 50 K. Subsequently, the positional restraints were removed, rototranslational constraints were introduced on all solute atoms [17], and the systems were further equilibrated each for 20 ps at 300 K. In the end, a simulation at a constant pressure of 1 atm was conducted for 300 ps.

After equilibration, production runs of 10 ns at constant pressure (1 atm) and temperature (300 K) were carried out. Pressure and temperature were kept constant using the weak-coupling scheme [18] with coupling times of 0.5 and 0.1 ps, respectively. The isothermal compressibility was set to 4.575 × 10^−4^ kJ^−1^ mol nm^3^, and two separate temperature baths were used for solute and solvent. The SHAKE algorithm was used to constrain bond lengths [19] allowing for 2-fs time-steps. Nonbonded interactions were calculated using a triple range scheme. Interactions within a short-range cutoff of 0.8 nm were calculated at every time step from a pair list that was updated every fifth step. At these points, interactions between 0.8 and 1.4 nm were also calculated explicitly and kept constant between updates. A reaction field [20] contribution was added to the electrostatic interactions and forces to account for a homogenous medium outside the long-range cutoff using a relative dielectric constant of 61 as appropriate for the SPC water model [21]. Coordinate and energy trajectories were stored every 0.5 ps for subsequent analysis.

### Conformational entropy calculations

Conformational entropy calculations were performed according to the formulation of Schlitter [22]:

1 $$S_{Schlitter} = \frac{1}{2} k_{B} \ln\det \left[ {\underline{\varvec{1}} + \frac{{k_{B} Te^{2} }}{{\hbar^{2} }}\underline{\varvec{M}}\underline{\varvec{\sigma}} } \right] $$

where *k*_B_ is Boltzmann’s constant, *T* the absolute temperature, *e* Euler’s number, ℏ Planck’s constant divided by 2π, ***M*** the 3*N*-dimensional diagonal matrix containing the N atomic masses of the solute atoms for which the entropy is calculated, and **σ** the covariance matrix of atom-positional fluctuations with the elements:

2 $$\sigma_{ij} = \left\langle {\left( {x_{i} - \left\langle {x_{i} } \right\rangle } \right)\left( {x_{j} - \left\langle {x_{j} } \right\rangle } \right)} \right\rangle $$

where *x*_i_ are the Cartesian coordinates of the atoms considered in the entropy calculation after a least-squares fit of the trajectory configurations using a particular subset of atoms.

## Results and discussion

### Structure of PDH

During all 10 ns MD simulations involving the protein, the root-mean-square deviation (RMSD) of the backbone atoms with respect to their initial crystal structure conformation remains below 0.3 nm, although for simulations NP_B, PP_A and PP_B the RMSD continues to rise, see Supplementary Content Fig. S1. Nevertheless, the observed RMSD values are low for all systems and indicate a stable protein backbone for all 10 ns MD simulations.

Secondary structure elements (DSSP) according to the Kabsch-Sander rules [23] for all simulations with PDH were assigned as a function of simulation time. Representative examples are compared in Supplementary Content Fig. S2. Overall, the secondary structure elements are very well conserved during the simulations. The most prominent differences in DSSP between the different MD simulations can be observed for amino acid residues 500–512 comprising Tyr-510, Val-511, and His-512, which all directly interact with GLC. The MD simulation of PDH shows a mixture between the β-bridge and the β-strand for these residues. In the complexes, the most prevalent secondary structure element for this region is either the β-bridge in e.g. system PP_A, simulation 2 or the β-strand in e.g. system PP_A, simulation 1. The manually inserted GLC has either slightly destabilizing or stabilizing effects on the PDH binding site and a clear trend cannot be identified.

**Fig. 2:**
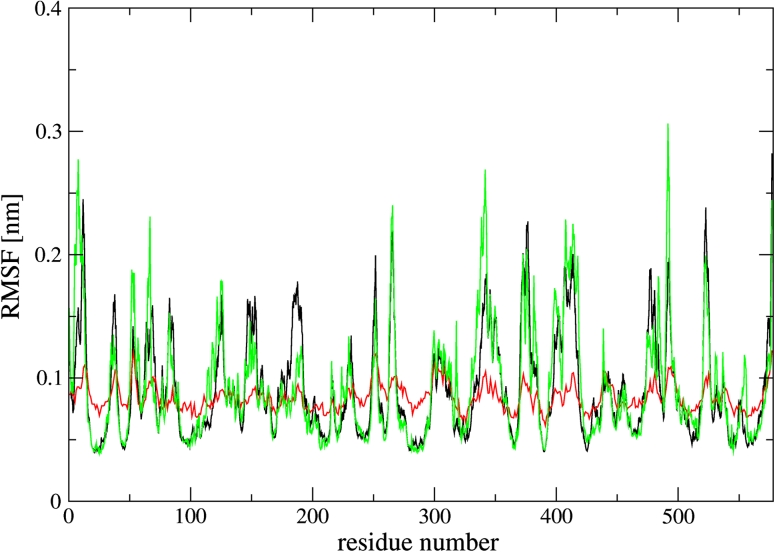
Root-mean-square fluctuations (RMSF) of the PDH backbone atoms. The RMSF values calculated from simulations PN_A, PN_B, NP_A, NP_B, PP_A, PP_B, were united according to the arithmetic mean (*black curve*) and compared to system PDH (*green curve*). RMSF values calculated from the crystal-structure B-factor values are depicted in *red*

Root-mean-square fluctuations (RMSF) of the PDH backbone were calculated over the 10 ns MD simulations (Fig. 2, Supplementary Content Fig. S3). As compared to the PDH run, all regions with high RMSF values for the complex simulations can be assigned to flexible solvent-exposed regions of the protein: Gln-145 to His-155, Asp-180 to Asn-190, Leu-345 to Asn-350, Lys-408 to Ala-415, and Met-473 to Lys-477. Note that the simulated RMSF are in good qualitative agreement with the RMSF calculated from the crystal-structure B-factor values (Fig. 2).

### Stability of the ligand, conformational entropies, and interaction energies

To investigate the thermodynamics of GLC binding, Schlitter ligand conformational entropies $S_{Schlitter}^{L}$ were calculated (Table 2). When fitted to GLC, $S_{Schlitter}^{L}$ for the complexes are lower compared to GLC simulated in water, indicating a loss of conformational entropy for GLC upon protein binding. The loss of conformational entropy upon binding is more pronounced for simulations PN_B and PP_A suggesting a more stable nature.

**Table 2:** Thermodynamics of binding between GLC and PDH in the various systems

System	$S_{Schlitter}^{L}$ ^a, b^	$S_{Schlitter}^{L}$ ^b, c^	$\left\langle {V_{ES}^{L - S} } \right\rangle$ ^d^	$\left\langle {V_{vdW}^{L - S} } \right\rangle$ ^d^
	J mol^−1^ K^−1^		kJ mol^−1^	
GLC	–	318	−285 ± 1	−9.5 ± 0.2
PN_A	430 ± 8	241 ± 17	−265 ± 4	−47 ± 1
PN_B	344 ± 35	211 ± 25	−215 ± 3	−73 ± 1
NP_A	412 ± 15	243 ± 4	−254 ± 3	−53 ± 1
NP_B	413 ± 37	249 ± 21	−288 ± 6	−47 ± 1
PP_A	340 ± 5	213 ± 6	−267 ± 2	−58 ± 1
PP_B	417 ± 37	253 ± 27	−253 ± 3	−57 ± 1

Schlitter $S_{Schlitter}^{L}$ ligand conformational entropies and average ligand-surrounding electrostatic $\left\langle {V_{ES}^{L - S} } \right\rangle$ as well as average ligand-surrounding van der Waals $\left\langle {V_{vdW}^{L - S} } \right\rangle$ interaction energies are reported as averages over individual simulation runs

^a^Calculation performed after a rototranslational fit on the protein backbone atoms

^b^Error estimates calculated as standard deviations between individual simulations

^c^Calculation performed after a rototranslational fit on d-glucose

^d^Error estimates calculated from block averaging [28] over individual runs and error propagation

In order to monitor the stability of GLC in the MD simulations of complex A and complex B, the root-mean-square deviation (RMSD) of the ligand was calculated with respect to its initial position after a translational and rotational fit of the protein backbone (Supplementary Content Fig. S4). The RMSD for GLC involving protonation states NP and PP remains predominantly below 0.25 nm. Larger deviations are observed for the PN protonation state and to some extent for simulations PP_B. To ensure that the increased mobility of GLC that was observed in these simulations was reproducible, two additional PP_B simulations were performed compared to the other systems, leading to very similar substrate mobilities.

The values of $S_{Schlitter}^{L}$ that were obtained after a conformational fit on the protein backbone (second column in table 2), do not only contain the conformational entropy, but also include the translational and rotational freedom of the substrate in the active site. Strikingly, these values confirm the stability of the substrate in systems PP_A, but also suggest a large structural stability for system PN_B. This suggests for the latter system that a stable pose was sampled, which was shifted by approximately 0.35–0.45 nm with respect to the initial structure.

The average electrostatic and van der Waals ligand-surrounding interaction energies of GLC in water and in the complex simulations are also listed in Table 2. The binding energies reveal that shape complementarity through nonpolar van der Waals interactions is the main driving force for GLC binding to PDH. This finding corroborates the experimentally observed promiscuity of the enzyme for a number of different sugar substrates [5]. In contrast, electrostatic contributions to binding between GLC and PDH are mostly unfavorable as indicated by higher average ligand–protein electrostatic interaction energies in complex A and complex B when compared to free GLC. However, electrostatics is still important for proper GLC orientation in the active site as indicated by the presence of different H-bonds in both complexes that will be discussed in detail in the subsequent section. A similar behavior was observed for the inhibition of extremely promiscuous cytochrome P450 enzymes [24, 25]. Strikingly, the most favorable van der Waals interaction energies correlate with the least favorable electrostatic interaction energies for system PN_B, for which a shift in position was previously deduced. Further, the electrostatic interaction energies seem very comparable in size for the fully protonated systems (PP) and the singly protonated systems (PN, NP).

### Important interactions between PDH and GLC

**Fig. 3:**
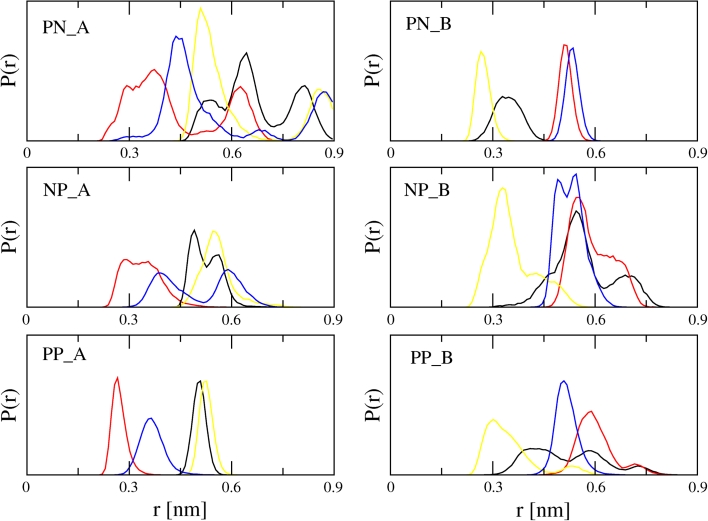
Normalized distance distributions between HC-atoms of d-glucose and the N5 atom of FAD over the combined PN_A, PN_B, NP_A, NP_B, PP_A, PP_B simulations. Coloring scheme: HC1–N5 (*black*), HC2–N5 (*red*), HC3–N5 (*yellow*), HC4–N5 (*blue*)

For the closely related GMC enzyme P2O, the reaction mechanism of the reductive half reaction involving a hydride transfer from atom C2 (GLC) to atom N5 (FAD) has been experimentally confirmed [26]. In order for such a hydride transfer to occur, the respective HC atom of GLC needs to be in the vicinity of the N5 atom of FAD. Therefore, the distances between atoms HC1–HC4 (GLC) and atom N5 (FAD) were monitored during the MD simulations of the complexes. Because GROMOS applies a united atom approach for methylidyne groups, virtual H-atoms were introduced to calculate the distance between the respective H-atom and N5. Figure 3 shows the distance distributions for the simulations. For complex A, the HC2–N5 distance is consistently the shortest and below a postulated cutoff of 0.3 nm in 15, 24 and 85 % of the time in systems PN_A, NP_A and PP_A, respectively. The distance HC4–N5 is shorter than 0.3 nm for short periods of time as well, while the distance for neither HC1–N5 nor HC3–N5 is ever below 0.3 nm. We therefore conclude that GLC oriented according to pose A will be most likely oxidized at the C2 position. Put differently, pose A represents the C2 oxidation mode of GLC with respect to the N5 atom of the FAD. For pose B, the HC3–N5 distance distribution is the shortest and is below the chosen 0.3 nm cutoff in 88, 14 and 22 % of the time in simulations PN_B, NP_ B and PP_B, respectively. The HC1–N5 distance also drops below this cutoff occasionally, while none of the other hydrogen atoms are close enough to the N5 atom of FAD for a hydride transfer to occur. Therefore, we conclude that GLC oriented according to pose B represents the C3 oxidation mode of GLC.

**Fig. 4:**
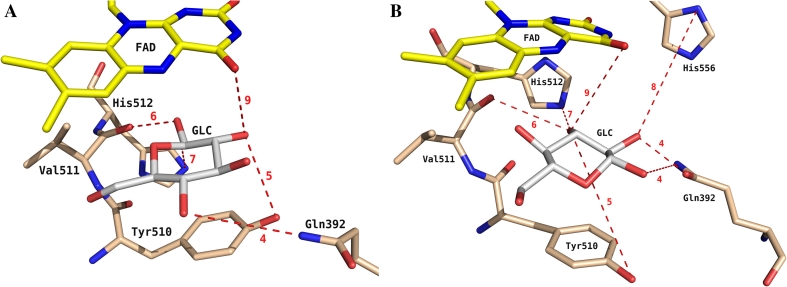
Two representative snapshots from MD trajectories of pose A (**a**) and pose B (**b**). Selected hydrogen bonds between d-glucose and active site amino acids are shown. Hydrogen bonds are labeled according to their ID in Table 3. Coloring scheme as in Fig. 1

The presence of hydrogen bonds was monitored utilizing a geometric criterion. A hydrogen bond was considered to be present if the hydrogen-acceptor distance was lower than 0.25 nm and the donor-hydrogen-acceptor angle was larger than 135°. Hydrogen bonds between PDH and GLC were monitored (Table 3; Fig. 4). The largest amount of overall substrate-protein hydrogen bonds is observed for system PN_B, which was previously seen to have the largest loss of conformational entropy, the least favorable electrostatic interaction energy and the most favorable van der Waals interaction energy. It forms prominent hydrogen bonds with the side chain of Gln-392, the backbone of Val-511 and the side chain of His-556. Residues Gln-392 and Val-511 are involved in (partially very strong) hydrogen bond interactions in the other systems as well. After system PN_B, systems PN_A and PP_A show the largest amount of hydrogen bonds. While this is due to a very strong interaction with the backbone of Val-511 for system PP_A, it is the result of a larger amount of shorter-lived hydrogen bonds for PN_A. This is again in agreement with the observed Schlitter entropies in Table 2; in system PP_A a single conformation is sampled, resulting in low entropy values, while system PN_A samples the active site more extensively, leading to higher entropy estimates. The versatile hydrogen-bonding pattern of systems PN_A, NP_A, NP_B and PP_B indicates once more the versatility of PDH in forming favorable interactions with different sugars.

**Table 3:** Occurrence of H-bonds between GLC and PDH, averaged over all simulations. In brackets, the interacting atoms in GLC are indicated

ID	Partner	PN_A (%)	PN_B (%)	NP_A (%)	NP_B (%)	PP_A (%)	PP_B (%)
1	Ser-64	12 (O6)					22 (O1)
2	Gly-105	40 (O3)					
3	Gly-359	13 (O4)					
4	Gln-392	21 (O2)	108 (O1/O5)	23 (O2)	24 (O2)	20 (O4)	62 (O1/O2)
5	Tyr-510	87 (O1/O2)		19 (O1)			33 (O3/O4)
6	Val-511		101 (O1/O2/O3)			181 (O1/O6)	29 (O3/O4)
7	His-512			32 (O1)	15 (O3)		
8	His-556		99 (O1/O2)		31 (O3)		
9	FAD	26 (O3)		31 (O2)	43 (O2/O3)		11 (O4)
	Total	199	308	105	113	201	157

A more detailed analysis of the observed hydrogen bonds offers an explanation for the experimental observation that the C2 product is not formed for the substrate methyl-α-d-glucopyranoside [27]. The methyl group at the O1 atom of the substrate removes the hydrogen bond donating capacity of this group. This reduces the total number of hydrogen bonds for systems PN_A, NP_A and PP_A by 63, 50 and 98 %, respectively, and for systems PN_B, NP_B and PP_B by 46, 0, and 34 %. Clearly, a loss of the hydrogen bond donating capacity of O1 in GLC destabilizes the C2 binding mode and leads only to C3 oxidation of methyl-α-d-glucopyranoside.

**Fig. 5:**
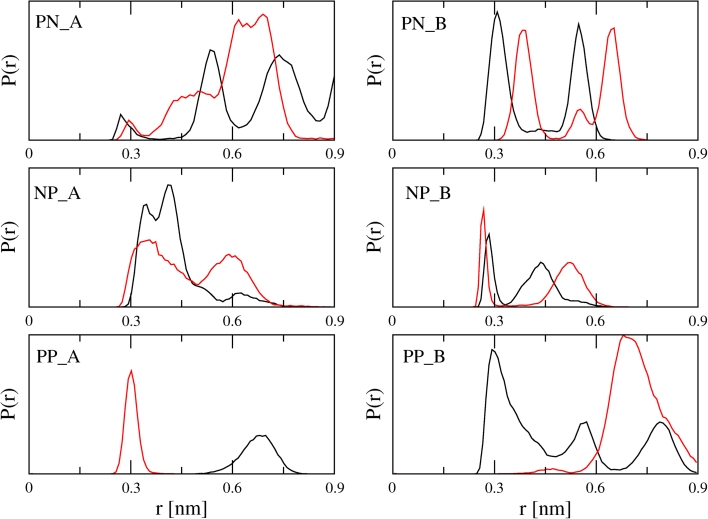
Normalized distributions of the shortest distance between N_δ_ or N_ε_ of His-512 (*black*) and His-556 (*red*) and d-glucose atom O2 in pose A (PN_A, NP_A, PP_A) and atom O3 in pose B (PN_B, NP_B, PP_B) in the combined simulations

In the reaction mechanism elucidated for C2 oxidation by P2O, a general base initially abstracts the C2-OH proton of GLC. In a second step, the protonated His-548 stabilizes the appearing alkoxide intermediate at C2-O^−^ and acts as the catalytic residue [26]. In contrast, PDH exhibits two histidines in the active site (His-512 and His-556). Therefore, we calculated the distance distributions from the N_δ_ or N_ε_ atoms (whichever was closer) of His-512 and His-556 to atoms O2 or O3 of GLC in pose A and pose B, respectively (Fig. 5). Again using a cutoff of 0.3 nm, it seems that both histidines can be positioned close enough to the hydroxyl groups to stabilize the deprotonation of the substrate. In systems PP_A and PP_B, the histidines both carry a positive charge and likely repel each other, leading to a more distinct preference for His-556 in pose A and for His-512 in pose B. Hydrogen bonds were observed between His-512 and His-556 in 35, 86, 78 and 68 % of the time for systems PN_A, PN_B, NP_A and NP_B, respectively. Being connected through hydrogen bonds, the exact protonation state is likely to interconvert quite easily. Accordingly, for these systems, both histidine residues are able to interact with the GLC hydroxyl groups (Fig. 5). Potentially, this offers another explanation for the substrate promiscuity of PDH. The ability of both histidines to take on the role of the catalytic base increases the versatility of the enzyme. In the system for which the GLC seems most stably anchored (PN_B), His-512 seems more likely to play the role of the catalytic residue. On the other hand, His-556 is involved in a stronger hydrogen bond interaction with the substrate. To a lesser extent, the opposite behavior is observed in system NP_A; although His-512 shows some hydrogen bonding with the substrate, His-556 comes slightly closer to atom O2, although a catalytic activity of His-512 cannot be excluded. Overall, we can conclude that His-512 samples distances that are in agreement with a catalytic function in all systems except for PP_A. His-556 is involved in more hydrogen bonds, but samples slightly less distances that are in agreement with a catalytic function. This observation is in agreement with the role of the two active site histidines His-502 and His-546 in aryl-alcohol oxidase (AAO, PDB: 3FIM) [29], another GMC member that is structurally similar to PDH [6]. In AAO, His-502 (corresponding to His-512 in PDH) plays a key role in the reductive half reaction acting as the catalytic base, whereas His-546 (corresponding to His-556 in PDH) has a more modest role, by correctly positioning the substrate through H-bonds during the reductive half reaction [29].

**Fig. 6:**
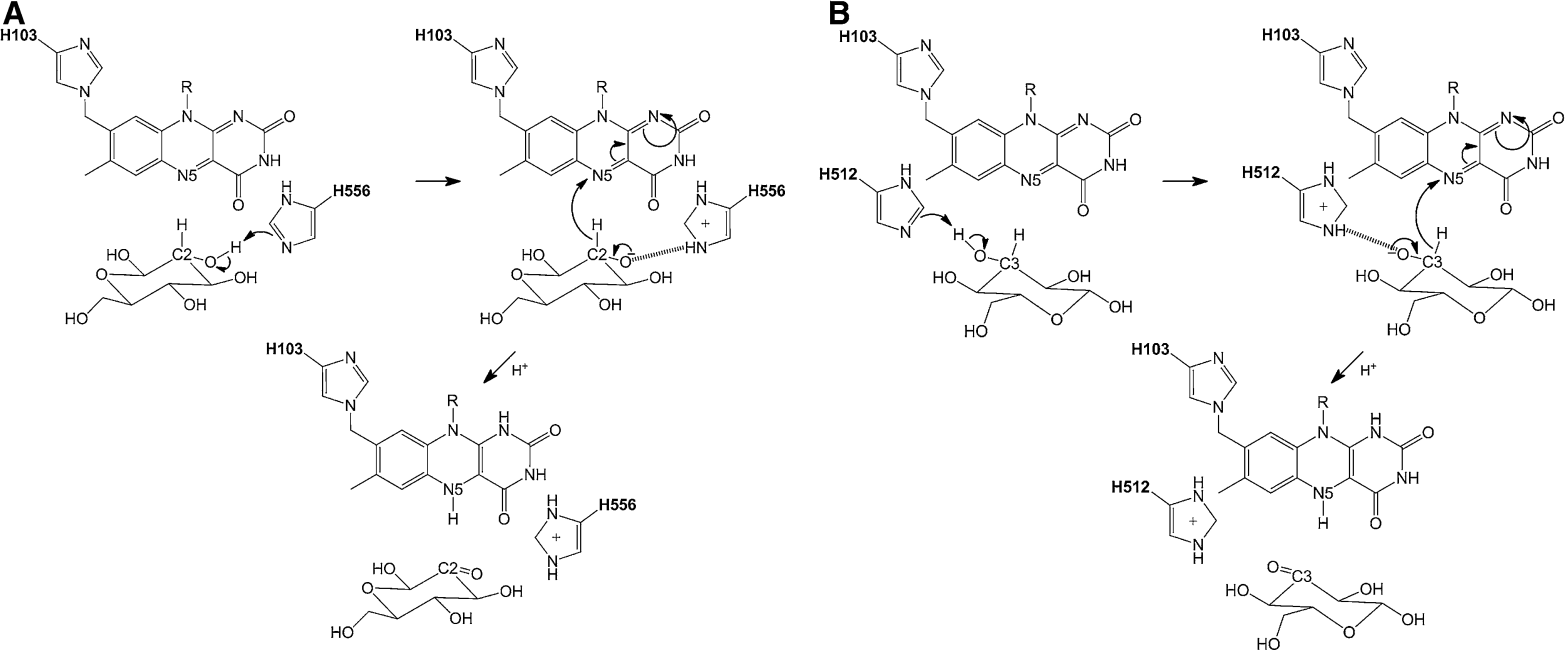
Proposed reaction mechanism for d-glucose oxidation at C2 in pose A (**a**) and at C3 in pose B (**b**). Both His-512 and His-556 can act as catalytic bases, with a slight preference for His-556 in the case of C2-oxidation (pose A) and for His-512 for C3-oxidation (pose B). The two suggested schemes are in agreement with the reaction mechanism of the reductive half-reaction of the structurally related P2O [26]

To conclude, Fig. 6 illustrates the proposed reaction mechanism of the first half reaction, corresponding to C2 and C3 oxidation of GLC. The two experimentally observed oxidation sites, corresponding to poses A and B in the simulations, can undergo deprotonation and subsequent stabilization involving both His-512 and His-556, as indicated in this figure.

## Conclusions

Extensive molecular dynamics simulations were performed to address the observed dioxidation of d-glucose at positions C2 and C3 by pyranose dehydrogenase as well as its promiscuous nature towards many different sugar substrates. RMSF analysis revealed good agreement between simulated and experimentally derived RMSF values. In addition, DSSP diagrams and RMSD values point toward a stable protein and GLC in all simulated systems providing additional confidence in the performed simulations.

To investigate the thermodynamics of GLC binding, the conformational entropies of GLC were calculated. Compared to GLC freely simulated in water, a loss of conformational entropy revealed an entropically unfavorable contribution to GLC binding for all complexes. Analysis of binding energies revealed that shape complementarity through nonpolar van der Waals interactions represents the main driving force for GLC binding, thereby corroborating the experimentally observed promiscuity of PDH. In contrast, electrostatic interactions were slightly unfavorable for GLC binding but they were still found to be important for proper GLC orientation within the active site through the hydrogen-bonding patterns that are observed.

A detailed hydrogen bond analysis offered an explanation for the absence of C2 product for the substrate methyl-α-d-glucopyranoside [27]: the methyl group at the O1 atom in this substrates prevents the donation of hydrogen bonds, which destabilizes the GLC C2 oxidation pose. Consequently, only pose B and the corresponding C3 oxidation product of GLC occurs.

Analysis of the distance distributions between GLC and active site histidines or the reactive N5 atom of FAD revealed insights into the proposed reaction mechanism. For pose A, the distances between HC2 (GLC) and N5 (FAD) were shortest and in agreement with a possible hydride transfer reaction. Based on the distances between the N-atoms in histidines 512 and 556 and the O2 atom in GLC, a slight preference for His-556 as the catalytic residue was observed, although His-512 could easily take over this function. For complex B, the distance between HC3 (GLC) and N5 (FAD) was shortest and a slight preference for His-512 as the catalytic residue was observed. These findings suggest that complex A represents the C2-oxidation mode, while complex B represents the C3-oxidation mode. The versatility of the enzyme is possibly enhanced by the presence of two histidine residues in the active site, which can both take on the role of the catalytic residue. To conclude, our data points to a similar reaction mechanism as previously reported for P2O [26]: oxidation of either C2 or C3 of GLC is accomplished through a proton abstraction by a general base and transition state stabilization by the active site His-556 or His-512, followed by a hydride transfer to atom N5 of FAD.

In summary, the promiscuity of PDH with respect to other GMC oxidoreductases can be attributed to various effects. Not only does PDH catalyze both C2 and C3 oxidation of GLC, while P2O only catalyzes C2 oxidation, PDH is also characterized by oxidation of a wide range of different carbohydrates. First, the active site offers various hydrogen bonding possibilities, such that alternative substrates or poses can be expected to find favorable interactions as well. Second, the actual binding is governed mostly by non-specific van der Waals interactions, allowing for the binding of many different substrates. Third, the presence of two active site histidines expands the versatility from the binding to the catalytic process itself. The related P2O enzyme does not only have a substrate loop, increasing the selectivity through specific interactions, it also lacks the second histidine residue observed in PDH.

As a final note, our work suggests various experiments that will be performed in the near future to characterize the function of several active site residues. Obviously, site directed mutagenesis on His-512 and His-556 may confirm our hypothesis that both residues may have a catalytic role. Further, based on the observed hydrogen bonding patterns, we suggest mutating Gln-392, Tyr-510 and Val-511, as these may influence the preferred product formation. Even though the interactions with Val-511 are through its backbone, a mutation to a more bulky, hydrophobic residue should be able to disrupt the observed hydrogen bonds. These findings indicate that MD simulations are indeed developing towards a standard tool from which experimentalists can draw ideas for new investigations.

## Electronic supplementary material

Below is the link to the electronic supplementary material.

Supplementary material 1 (PDF 937 kb)

## Abbreviations

AAO: Aryl-alcohol oxidase
CDH: Cellobiose dehydrogenase
DSSP: Secondary structure elements according to the Kabsch-Sander rules
FAD: Flavin adenine dinucleotide
GLC: d-glucose
GMC: Glucose–methanol–choline
GOX: Glucose oxidase
MD: Molecular dynamics
P2O: Pyranose 2-oxidase from *Trametes multicolor*
PDH: Pyranose dehydrogenase from *Agaricus meleagris*
RMSD: Root-mean-square deviation
RMSF: Root-mean-square fluctuation
$S_{Schlitter}^{L}$: Schlitter ligand conformational entropy

## References

[1] Sygmund C, Kittl R, Volc J, Halada P, Kubátová E, Haltrich D, Peterbauer CK (2008). Characterization of pyranose dehydrogenase from *Agaricus meleagris* and its application in the C-2 specific conversion of d-galactose. J Biotechnol.

[2] Volc J, Kubátová E, Wood DA, Daniel G (1997). Pyranose 2-dehydrogenase, a novel sugar oxidoreductase from the basidiomycete fungus *Agaricus bisporus*. Arch Microbiol.

[3] Volc J, Kubátová E, Daniel G, Sedmera P, Haltrich D (2001). Screening of basidiomycete fungi for the quinone-dependent sugar C-2/C-3 oxidoreductase, pyranose dehydrogenase, and properties of the enzyme from *Macrolepiota rhacodes*. Arch Microbiol.

[4] Kujawa M, Volc J, Halada P, Sedmera P, Divne C, Sygmund C, Leitner C, Peterbauer CK, Haltrich D (2007). Properties of pyranose dehydrogenase purified from the litter-degrading fungus *Agaricus xanthoderma*. FEBS J.

[5] Peterbauer CK, Volc J (2010). Pyranose dehydrogenases: biochemical features and perspectives of technological applications. Appl Microbiol Biotechnol.

[6] Tan T-C, Spadiut O, Wongnate T, Sucharitakul J, Krondorfer I, Sygmund C, Haltrich D, Chaiyen P, Peterbauer CK, Divne C (2013) The 1.6 Å Crystal structure of pyranose dehydrogenase from *Agaricus meleagris* rationalizes substrate specificity and reveals a flavin intermediate. PLoS ONE 8(1):e53567 10.1371/journal.pone.0053567 PMC3541233 23326459

[7] Leitner C, Volc J, Haltrich D (2001). Purification and characterization of pyranose oxidase from the white rot fungus *Trametes multicolor*. Appl Environ Microbiol.

[8] Kujawa M, Ebner H, Leitner C, Hallberg BM, Prongjit M, Sucharitakul J, Ludwig R, Rudsander U, Peterbauer CK, Chaiyen P, Haltrich D, Divne C (2006). Structural basis for substrate binding and regioselective oxidation of monosaccharides at C3 by pyranose 2-oxidase. J Biol Chem.

[9] Karplus M, Kuriyan J (2005). Molecular dynamics and protein function. Proc Natl Acad Sci USA.

[10] van Gunsteren WF, Bakowies D, Baron R, Chandrasekhar I, Christen M, Daura X, Gee P, Geerke DP, Glättli A, Hünenberger PH, Kastenholz MA, Oostenbrink C, Schenk M, Trzesniak M, van der Vegt NFA, Yu HB (2006). Biomolecular modeling: goals, problems, perspectives. Angew Chem Int Ed Engl.

[11] Tan T-C, Pitsawong W, Wongnate T, Spadiut O, Haltrich O, Chaiyen P, Divne C (2010). H-bonding and positive charge at the N(5)/O(4) locus are critical for covalent flavin attachment in *Trametes* pyranose 2-oxidase. J Mol Biol.

[12] Tan T-C, Haltrich D, Divne C (2011). Regioselective control of β-d-glucose oxidation by pyranose 2-oxidase is intimately coupled to conformational degeneracy. J Mol Biol.

[13] Blom N, Sicheritz-Pontén T, Gupta R, Gammeltoft S, Brunak S (2004). Prediction of post-translational glycosylation and phosphorylation of proteins from the amino acid sequence. Proteomics.

[14] Schmid N, Christ CD, Christen M, Eichenberger AP, van Gunsteren WF (2012). Architecture, implementation and parallelization of the GROMOS software for biomolecular simulation. Comp Phys Commun.

[15] Oostenbrink C, Villa A, Mark AE, Van Gunsteren WF (2004). A biomolecular force field based on the free enthalpy of hydration and solvation: the GROMOS force-field parameter sets 53A5 and 53A6. J Comput Chem.

[16] Berendsen HJC, Postma JPM, Van Gunsteren WF, Hermans J (1981). Interaction models for water in relation to protein hydration. Intermolecular forces.

[17] Amadei A, Chillemi G, Ceruso MA, Grottesi A, Di Nola A (2000). Molecular dynamics simulations with constrained roto-translational motions: theoretical basis and statistical mechanical consistency. J Chem Phys.

[18] Berendsen HJC, Postma JPM, van Gunsteren WF, DiNola A, Haak JR (1984). Molecular dynamics with coupling to an external bath. J Chem Phys.

[19] Ryckaert J-P, Ciccotti G, Berendsen HJ (1977). Numerical integration of the cartesian equations of motion of a system with constraints: molecular dynamics of n-alkanes. J Comp Phys.

[20] Tironi IG, Sperb R, Smith PE, van Gunsteren WF (1995). A generalized reaction field method for molecular dynamics simulations. J Chem Phys.

[21] Heinz TN, van Gunsteren WF, Hünenberger PH (2001). Comparison of four methods to compute the dielectric permittivity of liquids from molecular dynamics simulations. J Chem Phys.

[22] Schlitter J (1993). Estimation of absolute and relative entropies of macromolecules using the covariance matrix. Chem Phys Lett.

[23] Kabsch W, Sander C (1983). Dictionary of protein secondary structure: pattern recognition of hydrogen-bonded and geometrical features. Biopolymers.

[24] Vasanthanathan P, Olsen L, Jørgensen FS, Vermeulen NPE, Oostenbrink C (2010). Computational prediction of binding affinity for CYP1A2-ligand complexes using empirical free energy calculations. Drug Metab Dispos.

[25] Bren U, Oostenbrink C (2012). Cytochrome P450 3A4 inhibition by ketoconazole: tackling the problem of ligand cooperativity using molecular dynamics simulations and free-energy calculations. J Chem Inf Model.

[26] Wongnate T, Sucharitakul J, Chaiyen P (2011). Identification of a catalytic base for sugar oxidation in the pyranose 2-oxidase reaction. ChemBioChem.

[27] Volc J, Sedmera P, Halada P, Daniel G, Přikrylová V, Haltrich D (2002). C-3 oxidation of non-reducing sugars by a fungal pyranose dehydrogenase: spectral characterization. Mol Cat B.

[28] Allen MP, Tildesley DJ (1989). Computer simulation of liquids.

[29] Hernández-Ortega A, Lucas F, Ferreira P, Medina M, Guallar V, Martínez AT (2012). Role of active site histidines in the two half-reactions of the aryl-alcohol oxidase catalytic cycle. Biochemistry.

