# Supplementary material for: Molecular dynamics simulations give insight into d-glucose dioxidation at C2 and C3 by Agaricus meleagris pyranose dehydrogenase
Source: J Comput Aided Mol Des. 2013 Apr 17;27(4):295–304. doi: 10.1007/s10822-013-9645-7 (PMC3657087; doi:10.1007/s10822-013-9645-7)

Supplementary Content

Molecular dynamics simulations give insight into D-glucose dioxidation at C2 and C3 by *Agaricus meleagris* pyranose dehydrogenase

Michael M. H. Graf [a,1], Urban Bren [b,c,1], Dietmar Haltrich [a], Chris Oostenbrink [b,†]

[1] The first and second authors contributed equally to this work.

[a] Food Biotechnology Laboratory, Department of Food Science and Technology, University of Natural Resources and Life Sciences (BOKU), Muthgasse 18, AT-1190 Vienna, Austria.

[b] Institute of Molecular Modeling and Simulation, BOKU, University of Natural Resources and Life Sciences, Muthgasse 18, AT-1190 Vienna, Austria.

[c] Laboratory for Molecular Modeling, National Institute of Chemistry, Hajdrihova 19, SI-1001 Ljubljana, Slovenia.

† Corresponding author. Tel.: +43 1 47654 8302; fax: +43 1 47654 8309. E-mail address: chris.oostenbrink@boku.ac.at (C. Oostenbrink).

Supplementary Fig. S1 Root-mean-square deviation (RMSD) of the backbone during the 10 ns MD simulations of systems PN_A, PN_B, NP_A, NP_B, PP_A, PP_B and PDH. Coloring scheme: run 1 (black), run 2 (red), run 3 (green), run 4 (blue).

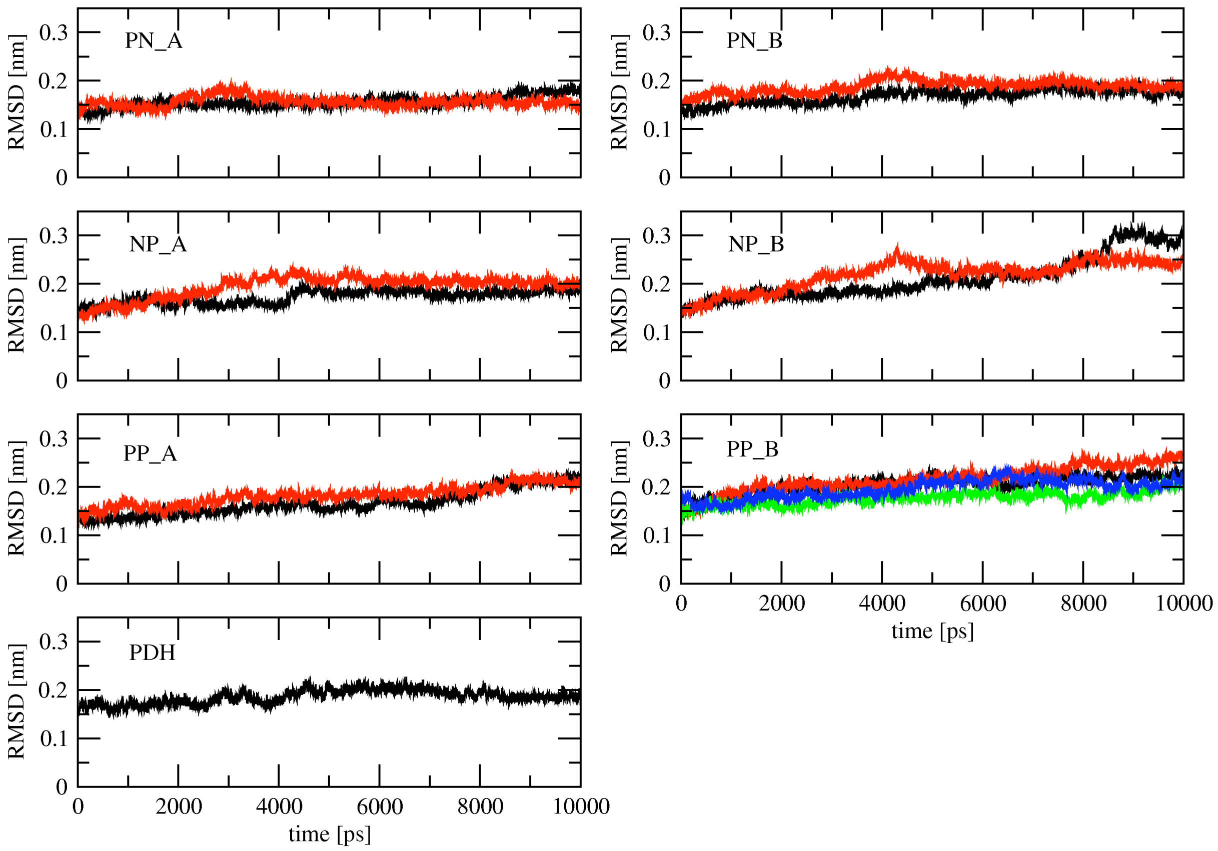

Supplementary Fig. S2 Secondary structure elements (DSSP) classification as a function of time for selected simulations.

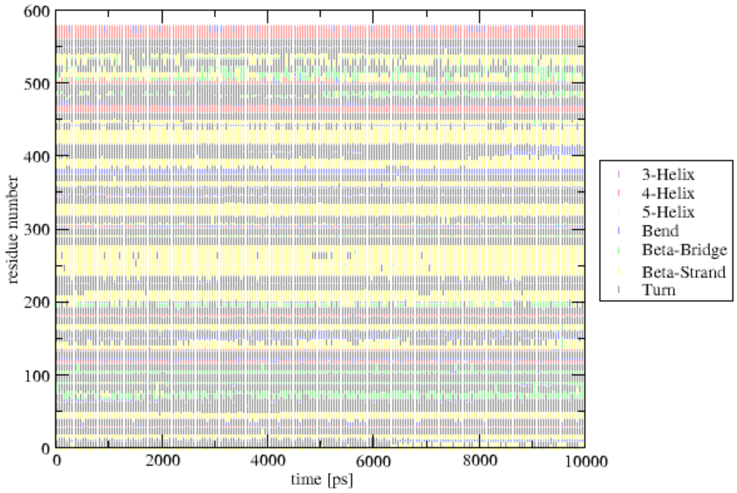

PDH

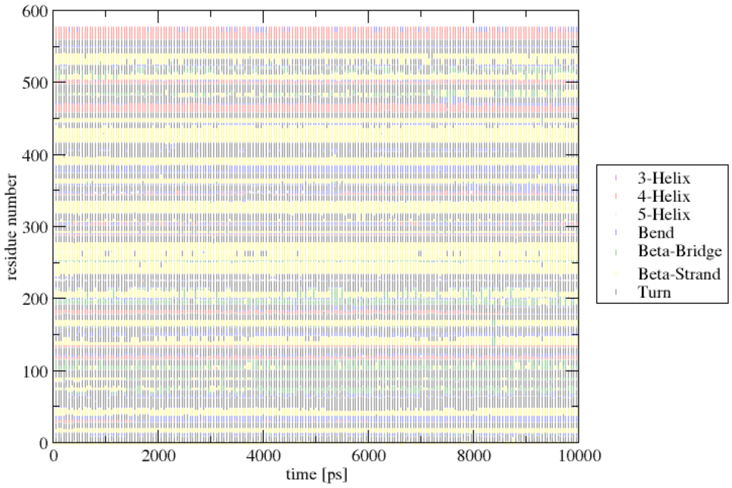

PP_A, run 1

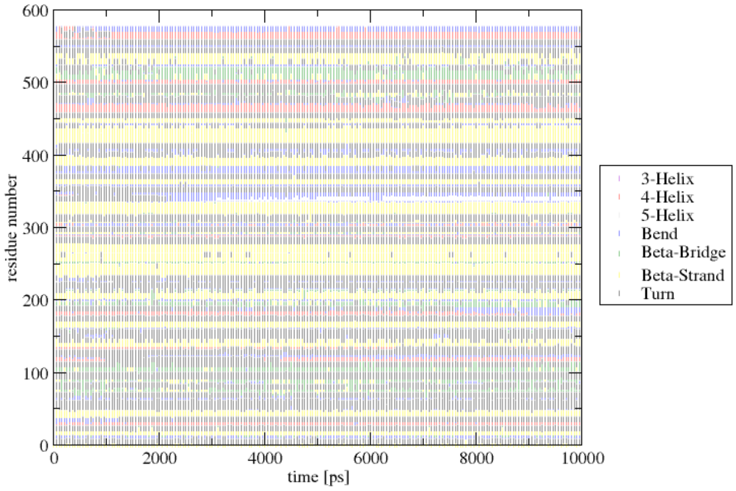

PP_A, run 2

Supplementary Fig. S3 Atom-positional root-mean-square fluctuations (RMSF) for the individual simulations. The average of the simulation curves yields the curve in figure 2 of the main manuscript. The RMSF values calculated from the crystal structure B-factor values are indicated with a thicker green curve.

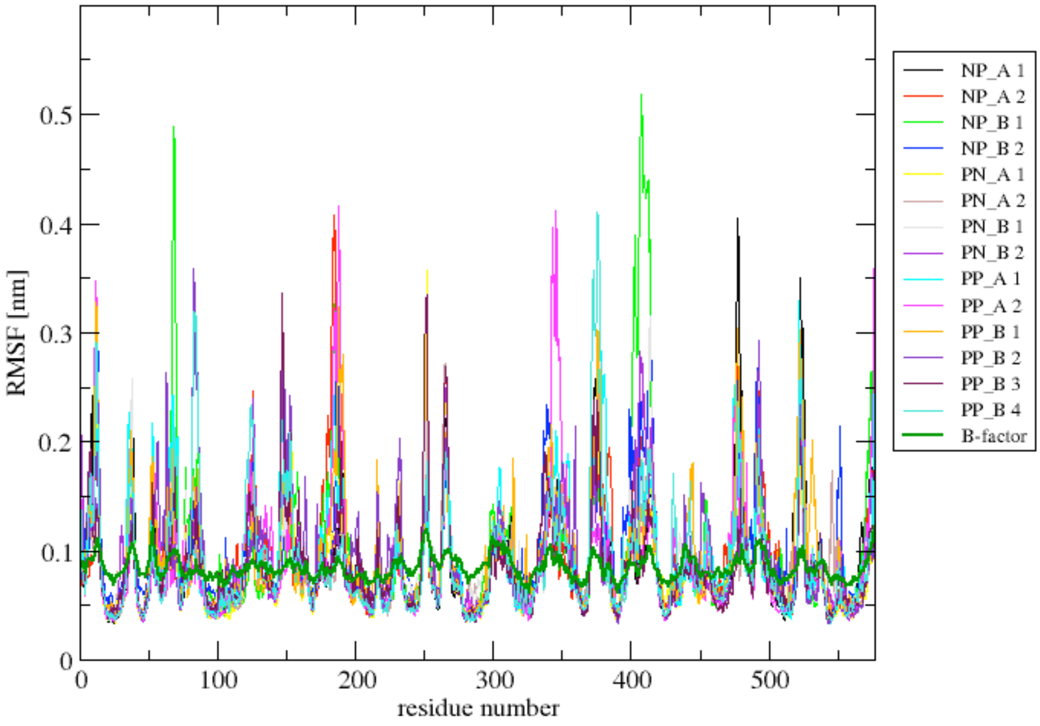

Supplementary Fig. S4 Normalized distributions of the atom-positional root-mean-square deviation (RMSD) of D-glucose after a roto-translational fit of the protein backbone with respect to the initial structure during the 10 ns MD simulations.

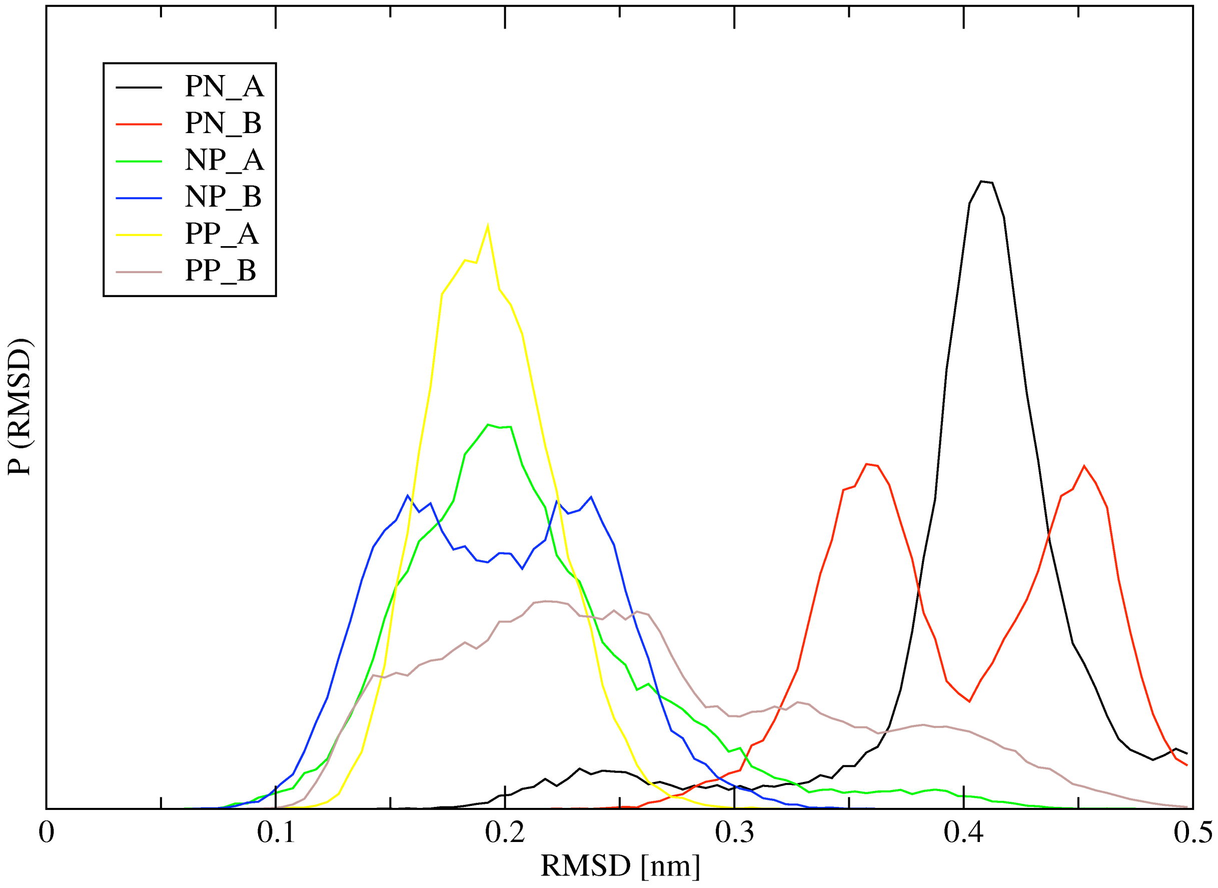

Supplement: Supplementary file 1 — Supplementary material 1 (PDF 937 kb) [file 10822_2013_9645_MOESM1_ESM.pdf]
